# Association between Unhealthy Dietary Habits and Proteinuria Onset in a Japanese General Population: A Retrospective Cohort Study

**DOI:** 10.3390/nu12092511

**Published:** 2020-08-19

**Authors:** Toshiaki Tokumaru, Tadashi Toyama, Akinori Hara, Kiyoki Kitagawa, Yuta Yamamura, Shiori Nakagawa, Megumi Oshima, Taro Miyagawa, Koichi Sato, Hisayuki Ogura, Shinji Kitajima, Yasunori Iwata, Norihiko Sakai, Miho Shimizu, Kengo Furuichi, Atsushi Hashiba, Takashi Wada

**Affiliations:** 1 Department of Nutrition, Kanazawa University Hospital, Kanazawa 9208641, Japan; tkmr-knz@staff.kanazawa-u.ac.jp; 2 Department of Nephrology and Laboratory Medicine, Kanazawa University, Kanazawa 9208641, Japan; yuta.yamamura@gmail.com (Y.Y.); yonedayone@gmail.com (S.N.); mgm_oshima@yahoo.co.jp (M.O.); ns50f1982@gmail.com (T.M.); sato.k@staff.kanazawa-u.ac.jp (K.S.); hisayuki.ogura@staff.kanazawa-u.ac.jp (H.O.); kitajimajima@yahoo.co.jp (S.K.); iwatay@staff.kanazawa-u.ac.jp (Y.I.); norin0826@staff.kanazawa-u.ac.jp (N.S.); mshimizu@staff.kanazawa-u.ac.jp (M.S.); furuichi@kanazawa-med.ac.jp (K.F.); twada@m-kanazawa.jp (T.W.); 3 Department of Environmental and Preventive Medicine, Kanazawa University, Kanazawa 9208640, Japan; ahara@m-kanazawa.jp; 4 Division of Internal Medicine, National Hospital Organization Kanazawa Medical Center, Kanazawa 9208650, Japan; kiyoki-knz@umin.org; 5 Department of Nephrology, Kanazawa Medical University School of Medicine, Kanazawa 9200293, Japan; 6 Kanazawa Medical Association, Kanazawa, 9200912, Japan; hashiba@kma.jp

**Keywords:** dietary habits, late dinner, skipping breakfast, proteinuria

## Abstract

The relationship between dietary habits and development of chronic kidney disease (CKD) is unclear. This retrospective cohort study was conducted to examine the association between unhealthy dietary habits and proteinuria onset, a key prognostic factor of CKD, among a Japanese general population aged ≥40 years. The risks of proteinuria onset were estimated based on the status of baseline unhealthy dietary habits (quick eating, late dinner, late evening snack, and skipping breakfast) compared with the status without these habits. A total of 26,764 subjects were included, with a mean follow-up period of 3.4 years. The most frequent unhealthy dietary habit was quick eating (29%), followed by late dinner (19%), late evening snack (16%), and skipping breakfast (9%). During the follow-up period, 10.6% of participants developed proteinuria. Late dinner and skipping breakfast showed an increased adjusted risk of proteinuria onset (hazard ratio (HR) 1.12, 95% confidence interval (CI) 1.02 to 1.22, and HR 1.15, 95% CI 1.01 to 1.31, respectively). Unhealthy dietary habits were not associated with changes in body mass index or waist-to-height ratio during the follow-up period. Our results suggest that late dinner and skipping breakfast are associated with higher risks for proteinuria onset.

## 1. Introduction

Chronic kidney disease (CKD) is defined as either a structural and/or functional abnormality of the kidney or a reduced glomerular filtration rate at <60 mL/min/1.73 m^2^ [1]. The incidence of end-stage renal disease is 287 per million person-years; the number of patients requiring renal replacement therapy continues to increase [2].

CKD is associated with increased risk for all-cause and cardiovascular mortality, which is a major health problem in several countries [3]. The major causes of CKD include diabetes [4], hypertension [4], and obesity [5], which are caused by poor eating habits. For example, skipping breakfast increases the risk of overweight or obesity by 48% [6]. The prevention and treatment of CKD include medication [7], smoking cessation [8], and dietary modifications. In general, diets include nutrient intake, nutrient balance, and dietary habits. For instance, sodium restriction, which involves modifying nutrient intake, is a well-known treatment for patients with CKD [9].

Studies conducted to assess the associations between diets and CKD have focused on nutrient intake or nutrient balance. For example, regarding nutrient intake, phosphorus restriction is effective against CKD-associated mineral and bone disorders in patients with CKD stages 3–5 [10]; regarding nutrient balance, a protein-restricted diet was found to be effective in preventing the progression of CKD [11]. In addition to nutrient intake and nutrient balance, dietary habits, including meal times and methods of eating such as the order of dishes, have received attention in recent years [12,13].

Dietary habits are known to be associated with lifestyle-related diseases. For instance, skipping breakfast is a risk factor for diabetes [14], and late dinner is associated with obesity [15]. Being modifiable risk factors, understanding these unhealthy dietary habits is important for the treatment of lifestyle-related diseases, such as diabetes or obesity [16]. Although unhealthy dietary habits causing metabolic disorders are potential risk factors for CKD, the relationship between dietary habits and CKD has not been fully clarified. Therefore, the present study was conducted to investigate the association between unhealthy dietary habits and proteinuria onset, which is a key prognostic factor of CKD [17].

## 2. Materials and Methods

### 2.1. Study Design and Subjects

We conducted a retrospective cohort study among a Japanese general population aged ≥40 years who underwent annual medical checkups from 1998 to 2014 in Kanazawa, Japan. The inclusion criteria were an estimated glomerular filtration rate (eGFR) at baseline of >60 mL/min/1.73 m^2^ and a follow-up period of more than 1 year. The exclusion criteria were a baseline urinary protein dipstick result of ≥1+ and the unavailability of the status of dietary habits or other covariates.

### 2.2. Measurements of Study Variables

Details regarding unhealthy dietary habits, daily drinking status, current smoking status, use of antihypertensive agents, and use of glucose-lowering medication were collected from the self-reported questionnaire used for the annual medical checkups. The body mass index (BMI) and waist-to-height ratio (WtHR) were recorded annually.

Unhealthy dietary habits were defined as follows: late dinner, i.e., eating dinner within 2 h of going to bed at a frequency of three or more times a week; skipping breakfast three or more times a week; quick eating, i.e., eating faster than people of the same age group; and late evening snack, i.e., eating snacks after dinner three or more times a week. Subjects chose either “yes” or “no” in each questionnaire item, except for quick eating and daily drinking. Participants were classified into the quick eating category when they chose “fast” from the list of “fast, ordinary, or slow”. They were classified into the daily drinking category when they answered “every day” from the list of “every day, sometimes, or rare”.

BMI was calculated by dividing body weight (kg) by the square of height (m). Waist circumference was measured at navel height in the standing position. WtHR, which better reflects visceral fat than waist circumference [18], was calculated by dividing waist circumference (cm) by height (cm). Blood pressure was measured at rest in the sitting position. The urine dipstick test was performed using random spot urine samples and recorded as negative, trace, 1+, 2+, 3+, or 4+; urine dipstick test 1+ corresponds to a proteinuria concentration of 30 mg/dL. eGFR was calculated from serum creatinine using the equation developed by the Japanese Society of Nephrology [19]. In the case of subjects taking antihypertensive agents, to obtain the potential blood pressure, we uniformly added 10 mmHg for systolic blood pressure (SBP) and 5 mmHg for diastolic blood pressure [20]. Diabetes was defined as HbA1c ≥ 6.5% (National Glycohemoglobin Standardization Program) and/or fasting blood glucose ≥ 7 mmol/L [21] or using glucose-lowering medication. Outcome was defined as the time to the onset of dipstick proteinuria ≥1+ during a follow-up period.

### 2.3. Statistical Analysis

Baseline data with normal distribution are reported as mean and standard deviation, skewed variables are represented as median and interquartile range, and categorical variables are shown as numbers and proportions. Baseline mean BMI and WtHR values [22,23], were calculated based on the presence or absence of each unhealthy dietary habit at baseline with adjustment for age, sex, and current smoking. Changes in BMI and WtHR, which were considered as possible intermediate factors in unhealthy dietary habits and proteinuria onset, were tested using a linear mixed-effects model with random intercept and random slope. For estimating proteinuria onset, we calculated the incidence rates per 1000 person-years based on the presence or absence of each unhealthy dietary habit, and Kaplan–Meier curves were also constructed. Multivariable Cox proportional hazards models were used to estimate the risks of proteinuria onset based on baseline unhealthy dietary habits. To evaluate the potential effect modification based on age, sex, and obesity, which are known as risk factors for CKD [22,24], we stratified the subjects according to age (<65 and ≥65 years), sex, and BMI (<25 and ≥25 kg/m^2^) and tested the interaction terms between unhealthy dietary habits and each of the variables age, sex, and BMI. In the multivariable analysis, each model was adjusted for the following potential confounders based on previous studies: age, sex, BMI, SBP, eGFR, hemoglobin, triglyceride, total cholesterol, HbA1c, serum uric acid, daily drinking, and current smoking [25]. A two-tailed significance level was set at *p* < 0.05. All analyses were performed using the Stata/IC statistical software (version 14; StataCorp LLC, College Station, TX, USA).

### 2.4. Ethics Statement

The study protocol was approved by the ethics committee of Kanazawa University Hospital (approval number: 2287-1). All data were collected and de-identified by the Kanazawa Medical Association. The study was conducted in accordance with the Declaration of Helsinki.

## 3. Results

### 3.1. Baseline Characteristics of Subjects

**Figure 1:**
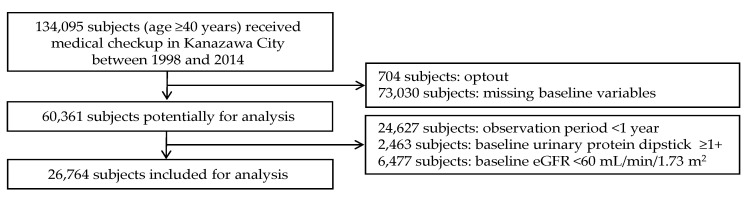
Flow diagram of the study. Baseline variables included unhealthy dietary habits (late dinner, skipping breakfast, quick eating, and late evening snack), height, body weight, waist circumference, blood pressure, urine protein (dipstick measurement), serum creatinine, hemoglobin, triglyceride, total cholesterol, blood glucose, glycated hemoglobin (HbA1c), serum uric acid, daily drinking, current smoking, use of antihypertensive agents, and use of glucose-lowering medication. eGFR, estimated glomerular filtration rate.

Figure 1 shows the flow diagram of the selection of study subjects. Among subjects who underwent a medical checkup between 1998 and 2014, 26,764 subjects met the inclusion criteria and were included in the analysis.

Table 1 shows the baseline characteristics of the study participants. Mean age was 68 years, and 44% of the participants were men. The most frequent unhealthy dietary habits were quick eating (29%), followed by late dinner (19%), late evening snack (16%), and skipping breakfast (9%). The mean follow-up period was 3.4 years. Table S1 (Supplementary Materials) shows the baseline characteristics of the participants according to unhealthy dietary habits.

### 3.2. Changes in BMI and WtHR According to Baseline Unhealthy Dietary Habits

**Figure 2:**
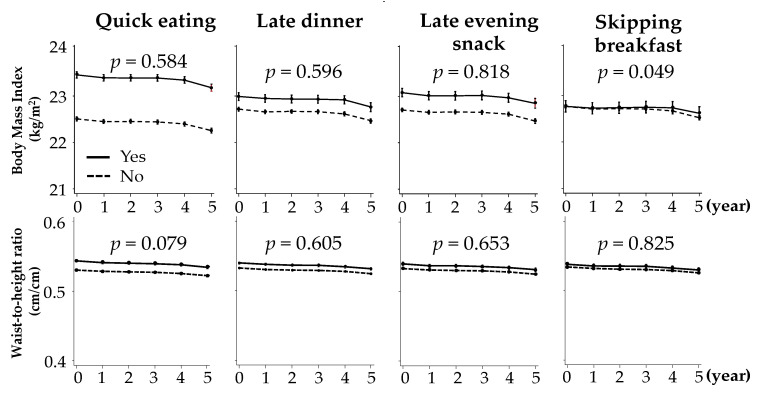
Changes in body mass index and waist-to-height ratio according to baseline unhealthy dietary habits. Adjusted for age, sex, body mass index, systolic blood pressure, estimated glomerular filtration rate, hemoglobin, triglyceride, total cholesterol, HbA1c, serum uric acid, daily drinking, and current smoking. Error bars represent the 95% confidence interval.

Adjusted baseline BMI and WtHR values were higher in participants with each of the unhealthy dietary habits Table S2 (Supplementary Materials). Both adjusted mean BMI and adjusted mean WtHR decreased during the five-year follow-up period for each unhealthy dietary habit. Participants with a habit of skipping breakfast exhibited a slightly slower decline in BMI during the follow-up period (*p* = 0.049). The other dietary habits were not associated with changes in BMI or WtHR (Figure 2).

### 3.3. Unhealthy Dietary Habits and Risks for Proteinuria Onset

During the follow-up period, 10.6% of the participants developed proteinuria, with an incidence rate of 32.7 per 1000 person-years. The number, person-years of follow-up, event rates, and hazard ratio of proteinuria onset for each unhealthy dietary habit are shown in Table 2 and Table S3 (Supplementary Materials).

**Figure 3:**
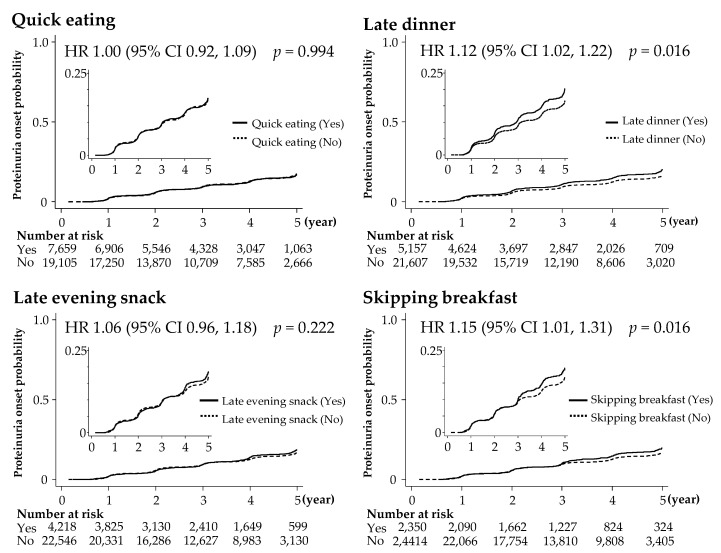
Kaplan–Meier curves and hazard ratio of proteinuria onset for each unhealthy dietary habit. Hazard ratios were adjusted for age, sex, body mass index, systolic blood pressure, estimated glomerular filtration rate, hemoglobin, triglyceride, total cholesterol, HbA1c, serum uric acid, daily drinking, and current smoking. The insets show the same data on enlarged y-axes. HR, hazard ratio; CI, confidence interval.

**Figure 4:**
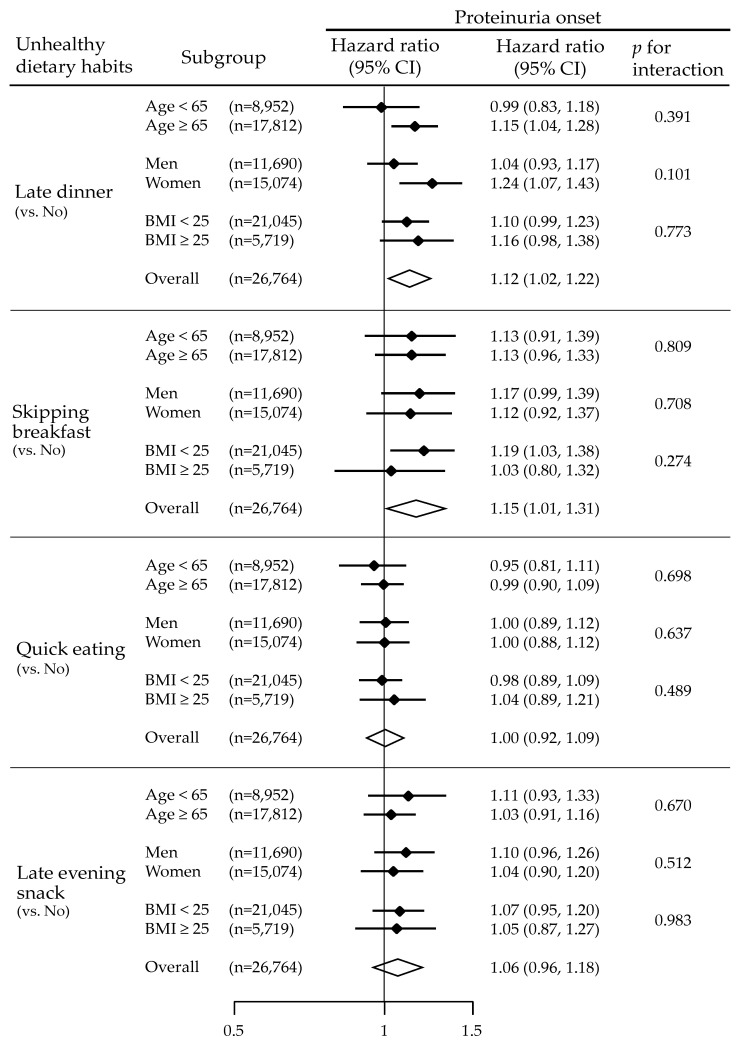
Association between unhealthy dietary habits and proteinuria onset according to the baseline categories of age, sex, and body mass index. Adjusted for age, sex, body mass index, systolic blood pressure, estimated glomerular filtration rate, hemoglobin, triglyceride, total cholesterol, HbA1c, serum uric acid, daily drinking, and current smoking. (When the variable of subgroup was included in the explanatory variable, it was excluded from adjustment.).

Figure 3 shows the Kaplan–Meier curves and the hazard ratio of proteinuria onset for each unhealthy dietary habit. Late dinner and skipping breakfast demonstrated significantly higher risks for proteinuria onset after multivariable adjustments (*p* = 0.016 and *p* = 0.016, respectively). To confirm the interaction of late dinner and skipping breakfast, we added a variable multiplied by late dinner and skipping breakfast to the model and analyzed it, but an interaction between late dinner and skipping breakfast was not observed (*p* = 0.222). The habits of quick eating and late evening snack were not associated with increased risks for proteinuria onset (*p* = 0.994 and *p* = 0.222, respectively). The interactions between baseline dietary habits and each of the baseline variables, i.e., age (<65 and ≥65 years), sex, and BMI (<25 and ≥25 kg/m^2^), were not significant for any type of unhealthy dietary habit (Figure 4).

## 4. Discussion

In this retrospective study of a Japanese general population, we investigated the association between unhealthy dietary habits and proteinuria onset. We found that late dinner and skipping breakfast were associated with higher risks for proteinuria onset. This result was significant even after adjusting for potential confounders, such as BMI and WtHR.

There are a variety of definitions of eating habits that must be considered when comparing them with previous studies. For example, the participants in our study showed lower proportions of late dinner and skipping breakfast than those in previous studies. A study of South Asian Canadians defined late dinners as after 8 p.m., which was found in 37% of the subjects [26]. In another study of Americans, skipping breakfast was defined as when the meal was taken, and the proportions were 17% for men and 24% for women [27,28]. In addition to the differences in the questionnaire, the higher mean age in our study might have resulted in the lower proportions of unhealthy dietary habits than those in other studies.

Although lifestyle is an important factor in reducing residual risk, when compared with established interventions such as antihypertensive therapy and smoking cessation, each lifestyle is characterized by a close relationship to the established interventions. In an observational study, Katsuma et al. reported that late dinner and skipping breakfast might have an interaction for risks of proteinuria [15]. In our study, a status of having both late dinner and skipping breakfast habits showed no increased risk for proteinuria. The reason for this difference is unclear, but it might be due to the different questionnaire or the definitions of unhealthy dietary habits.

Dietary habits, such as late dinner and skipping breakfast, were reported to be associated with obesity or increased waist circumference. For instance, in an observational study, Sakurai et al. reported that skipping breakfast was closely associated with annual changes in BMI and waist circumference among men [29]. In another longitudinal study, late dinner was associated with obesity [30].

The mean body weight decreased in the study participants during the follow-up period. Because body weight loss is associated with stable kidney function and reduced albuminuria [31], we may not have adequately investigated the association between baseline body weight and the onset of proteinuria. It is uncertain why BMI and WtHR did not increase in people with unhealthy dietary habits in our study; however, the findings implied that they might not be intermediate factors in our study setting. The possibility that weight loss may not serve as an appropriate surrogate for the reduction in the risk of CKD is a point of caution when providing dietary guidance to high-risk individuals.

In this study, we confirmed the importance of these dietary habits and proteinuria onset in a large-scale Japanese general population. Previous studies examined dietary habits and risks for kidney injury. In a cross-sectional study, Fujibayashi et al. reported that eating irregular meals was associated with a 1.40-times-higher risk for proteinuria [32]. Another observational study reported that people having two unhealthy lifestyles have a 2.04-times-higher risk for CKD [33]. The effect sizes associated with dietary habits in our study were slightly smaller compared to those associated with dietary habits in the previous studies, but they are important for contributing to the reduction of residual risk.

In our study setting, factors affecting dietary habits and the development of proteinuria were uncertain, but unobserved hypertension may have been a possible intermediate variable. For instance, physiological suppression of cortisol at night is less observed after late dinner [34], and nocturnal cortisol elevation was reported to be associated with morning hypertension [35]. These mechanisms might be involved in the relationship between late dinner and proteinuria onset. With regard to skipping breakfast and hypertension, Witbracht et al. reported that skipping breakfast can increase blood pressure due to hunger stress [36]. Conversely, Ahuja et al. reported that eating breakfast can reduce the rise in blood pressure [37]. A study observing night-time blood pressure and blood pressure in the presence or absence of breakfast might be useful for examining the relationship between late dinner, skipping breakfast, and proteinuria onset. Although interactions were not significant, higher risk for proteinuria was observed in women with late dinner than men. The effects of short-term metabolic outcome related to late dinner do not differ between men and women [38], but risk factors for proteinuria onset might have developed in women with late dinner during the follow-up period.

Our study has several limitations. First, we could not include the changes in unhealthy dietary habits during the follow-up period. Second, our questionnaire could not evaluate supplementation of macro- and micronutrients and nutrient imbalance, which is associated with unhealthy dietary habits. Third, renin–angiotensin system inhibitors suppress proteinuria [39], but we were unable to include classes of antihypertensive agents in the model. Fourth, data on certain important confounding factors were unavailable; for example, work timing may impact meal timing and physical activity might be associated with proteinuria onset; however, both variables were not assessed. Fifth, the subjects might have received nutritional guidance during the study period, which was not recorded in our study.

## 5. Conclusions

Late dinner and skipping breakfast might be associated with proteinuria onset irrespective of baseline or changes in BMI and WtHR. Considering the aspect of preventive care for the general population, these modifiable dietary habits are important. To better use the results of this study for nutritional guidance, we recommend that dietary habits should be included and evaluated in multifactorial intervention studies like J-DOIT3 (Japan Diabetes Optimal Integrated Treatment study for 3 major risk factors of cardiovascular diseases) [40].

## Figures and Tables

**Table 1:** Baseline characteristics of participants.

Variables	All
N	26,764
Age, year	68 (9)
Men, *n* (%)	11,690 (44)
Body mass index, kg/m^2^	22.8 (3.1)
Waist circumference, cm	83 (9)
Waist-to-height ratio, cm/cm	0.53 (0.06)
Systolic blood pressure, mmHg	132 (18)
Diastolic blood pressure, mmHg	77 (11)
eGFR, mL/min/1.73 m^2^	77 (12)
Hemoglobin, g/dL	13.6 (1.4)
Triglyceride, mg/dL	103 (74, 146)
Total cholesterol, mg/dL	204 (33)
HbA1c, %	5.2 (5.0, 5.5)
Quick eating (yes), *n* (%)	7659 (29)
Late dinner (yes), *n* (%)	5157 (19)
Late evening snack (yes), *n* (%)	4218 (16)
Skipping breakfast (yes), *n* (%)	2350 (9)
Daily drinking (yes), *n* (%)	9225 (34)
Current smoking (yes), *n* (%)	3792 (14)

Data are presented in numbers (%), mean (SD), or median (interquartile range). eGFR, estimated glomerular filtration rate.

**Table 2:** Association between unhealthy dietary habits and hazard ratio of proteinuria onset.

	**Overall**			
Number of events	2844 (10.6%)			
Person-years	86,903			
Events per 1000 person-years	32.7			
	**Quick Eating**		**Late Dinner**	
	Yes	No	Yes	No
Number of events	805 (10.5%)	2039 (10.7%)	625 (12.1%)	2219 (10.3%)
Person-years	24,882	62,020	16,582	70,321
Events per 1000 person-years	32.4	32.9	37.7	32.5
Unadjusted HR (95% CI; *p*-value) (vs. No)	0.98 (0.91, 1.07; *p* = 0.711)		1.19 (1.09, 1.30; *p* < 0.001)	
	**Late Evening Snack**		**Skipping Breakfast**	
	Yes	No	Yes	No
Number of events	469 (11.1%)	2375 (10.5%)	265 (11.3%)	2579 (10.6%)
Person-years	13,783	73,120	7314	79,589
Events per 1000 person-years	34.0	32.5	36.2	32.4
Unadjusted HR (95% CI; *p*-value) (vs. No)	1.04 (0.95, 1.15; *p* = 0.396)		1.12 (0.99, 1,27; *p* = 0.078)	

HR, hazard ratio. CI, confidence interval. Adjusted for age, sex, body mass index, systolic blood pressure, estimated glomerular filtration rate, hemoglobin, triglyceride, total cholesterol, HbA1c, serum uric acid, daily drinking, and current smoking.

## Supplementary Materials

The following are available online at https://www.mdpi.com/2072-6643/12/9/2511/s1, Supplementary Table S1: Baseline characteristics of participants according to unhealthy dietary habits. Supplementary Table S2: Baseline mean body mass index and waist-to-height ratio according to unhealthy dietary habits. Supplementary Table S3: Hazard ratios of proteinuria onset for late dinner or skipping breakfast along with covariates.
