# Supplementary material for: Association between Unhealthy Dietary Habits and Proteinuria Onset in a Japanese General Population: A Retrospective Cohort Study
Source: Nutrients. 2020 Aug 19;12(9):2511. doi: 10.3390/nu12092511 (PMC7551801; doi:10.3390/nu12092511)
Supplement: Supplementary file 1 [file nutrients-12-02511-s001.pdf]

Supplementary Table S1. Baseline characteristics of participants according to unhealthy dietary habits

Variables	All	Quick eating		Late dinner		Late evening snack		Skipping breakfast	
		Yes	No	Yes	No	Yes	No	Yes	No
N (%)	26,764	7,659 (29)	19,105 (71)	5,157 (19)	21,607 (81)	4,218 (16)	22,546 (84)	2,350 (9)	24,414 (91)
Age, year	68 (9)	66 (9)	69 (9)	67 (10)	68 (9)	67 (10)	68 (9)	67 (10)	68 (9)
Men, n (%)	11,690 (44)	3,313 (43)	8,377 (44)	2,925 (57)	8,765 (41)	1,644 (39)	10,046 (45)	1,117 (48)	10,573 (43)
Body mass index, kg/m^2	22.8 (3.1)	23.5 (3.2)	22.5 (3.0)	23.1 (3.2)	22.7 (3.1)	23.0 (3.3)	22.7 (3.1)	22.8 (3.3)	22.7 (3.1)
Waist circumference, cm	83 (9)	85 (9)	82 (9)	84 (9)	83 (9)	83 (9)	84 (10)	83 (10)	83 (9)
Waist-to-height ratio, cm/cm	0.53 (0.06)	0.54 (0.06)	0.53 (0.06)	0.53 (0.06)	0.53 (0.06)	0.53 (0.07)	0.53 (0.06)	0.53 (0.06)	0.53 (0.06)
Systolic blood pressure, mmHg	132 (18)	133 (18)	132 (18)	133 (18)	132 (18)	131 (18)	133 (18)	132 (19)	132 (18)
Diastolic blood pressure, mmHg	77 (11)	78 (11)	77 (11)	78 (11)	77 (11)	77 (11)	78 (11)	78 (11)	77 (11)
eGFR, mL/min/1.73 m^2	77 (12)	78 (12)	77 (12)	79 (13)	77 (12)	78 (12)	78 (12)	80 (13)	77 (12)
Hemoglobin, g/dL	13.6 (1.4)	13.7 (1.4)	13.5 (1.4)	13.8 (1.5)	13.5 (1.4)	13.5 (1.4)	13.6(1.4)	13.7 (1.4)	13.5 (1.4)
Triglyceride, mg/dL	103 (74, 146)	106 (76, 152)	101 (73, 144)	103 (74, 149)	103 (74, 145)	103 (74, 149)	103 (74, 146)	106 (74, 154)	103 (74, 146)
Total cholesterol, mg/dL	204 (33)	206 (33)	204 (34)	202 (34)	205 (33)	207 (34)	204 (33)	206 (36)	204 (33)
HbA1c, %	5.2 (5.0, 5.5)	5.2 (5.0, 5.5)	5.2 (5.0, 5.5)	5.2 (5.0, 5.5)	5.2 (5.0, 5.5)	5.2 (5.0, 5.5)	5.2 (5.0, 5.5)	5.1 (4.9, 5.4)	5.2 (5.0, 5.5)
Serum uric acid, mg/dL	5.0 (1.3)	5.1 (1.3)	5.0 (1.3)	5.2 (1.3)	5.0 (1.2)	4.9 (1.3)	5.0 (1.3)	5.2 (1.4)	5.0 (1.3)
Daily drinking, n (%)	9,225 (34)	2,709 (35)	6,516 (34)	2,681 (52)	6,544 (30)	1,135 (27)	8,090 (36)	1,011 (43)	8,214 (34)
Current smoking, n (%)	3,792 (14)	1,152 (15)	2,640 (14)	1,147 (22)	2,645 (12)	657 (16)	3,135 (14)	743 (32)	3,050 (12)

Data are presented in numbers (%), mean (SD), or median (interquartile range). eGFR, estimated glomerular filtration rate

Supplementary Table S2. Baseline mean body mass index and waist-to-height ratio according to unhealthy dietary habits

Unhealthy dietary habits	Body mass index (kg/m^2)			Waist-to-height ratio (cm/cm)		
	Yes	No	*p*-value	Yes	No	*p*-value
Quick eating	23.3	22.5	< 0.001	0.54	0.53	< 0.001
Late dinner	23.0	22.7	< 0.001	0.54	0.53	< 0.001
Late evening snack	23.1	22.7	< 0.001	0.54	0.53	< 0.001
Skipping breakfast	22.8	22.7	0.788	0.53	0.53	0.018

Adjusted for age, sex, systolic blood pressure, estimated glomerular filtration rate, hemoglobin, triglyceride, total cholesterol, HbA1c, serum uric acid, daily drinking, and current smoking

Supplementary Table S3. Multivariable adjusted hazard ratios for proteinuria onset, caused by consuming dinner late or skipping breakfast, along with covariates

Late dinner and proteinuria onset				Skipping breakfast and proteinuria onset			
**Variables**	**Hazard ratio**	**(95% CI)**	***p*-value**	**Variables**	**Hazard ratio**	**(95% CI)**	***p*-value**
Late dinner (vs. no late dinner)	1.12	(1.02, 1.22)	0.016	Skipping breakfast (vs. no skipping breakfast)	1.15	(1.01, 1.31)	0.032
Age (+1 year)	1.02	(1.01, 1.02)	<0.001	Age (+1 year)	1.02	(1.01, 1.02)	<0.001
Men (vs. women)	1.53	(1.38, 1.69)	<0.001	Men (vs. women)	1.54	(1.39, 1.70)	<0.001
Body mass index (+1 kg/m^2)	1.02	(1.01, 1.03)	0.001	Body mass index (+1 kg/m^2)	1.02	(1.01, 1.04)	0.001
Systolic blood pressure (+5 mm/Hg)	1.05	(1.04, 1.06)	<0.001	Systolic blood pressure (+5 mm/Hg)	1.05	(1.04, 1.06)	<0.001
eGFR (-10 mL/min/1.73 m^2)	1.03	(0.99, 1.06)	0.135	eGFR (-10 mL/min/1.73m^2)	1.02	(0.99, 1.06)	0.139
Hemoglobin (-1 g/dL)	1.08	(1.04, 1.11)	<0.001	Hemoglobin (-1 g/dL)	1.08	(1.04, 1.11)	<0.001
Triglyceride (+10 mg/dL)	1.01	(1.00, 1.01)	0.005	Triglyceride (+10 mg/dL)	1.01	(1.00, 1.01)	0.005
Total cholesterol (+10 mg/dL)	0.98	(0.97, 0.99)	0.005	Total cholesterol (+10 mg/dL)	0.98	(0.97, 0.99)	0.004
HbA1c (+1 %)	1.20	(1.15, 1.26)	<0.001	HbA1c (+1 %)	1.20	(1.15, 1.26)	<0.001
Serum uric acid (+1 mg/dL)	0.99	(0.95, 1.02)	0.398	Serum uric acid (+1 mg/dL)	0.98	(0.95, 1.02)	0.375
Daily drinking (vs. no daily drinking)	0.94	(0.86, 1.02)	0.140	Daily drinking (vs. no daily drinking)	0.95	(0.87, 1.03)	0.215
Current smoking (vs. no current smoking)	1.26	(1.13, 1.40)	<0.001	Current smoking (vs. no current smoking)	1.25	(1.13, 1.39)	<0.001

CI, confidence interval; eGFR, estimated glomerular filtration rate
Adjusted for age, sex, body mass index, systolic blood pressure, estimated glomerular filtration rate, hemoglobin, triglyceride, total cholesterol, HbA1c, serum uric acid, daily drinking, and current smoking. Variables were mutually adjusted.
